# Improving the performance of a triage scale for chest pain patients admitted to emergency departments: combining cardiovascular risk factors and electrocardiogram

**DOI:** 10.1186/s12873-022-00680-y

**Published:** 2022-07-04

**Authors:** Chiara Casarin, Anne-Sophie Pirot, Charles Gregoire, Laurence Van Der Haert, Patrick Vanden Berghe, Diego Castanares-Zapatero, Melanie Dechamps

**Affiliations:** 1 Emergency Department, Saint-Luc University Hospital, Université Catholique de Louvain (UCLouvain), Brussels, Belgium; 2 Institute of Neuroscience (IoNS), Institut de Recherche Expérimentale et Clinique (IREC), UCLouvain, Brussels, Belgium; 3 Cardiovascular Intensive Care Unit, Saint-Luc University Hospital, UCLouvain, Brussels, Belgium; 4 Intensive Care Unit, Saint-Luc University Hospital, UCLouvain, Brussels, Belgium; 5 Pôle de Recherche Cardiovasculaire (CARD), Institut de Recherche Expérimentale et Clinique (IREC), UCLouvain, Brussels, Belgium

**Keywords:** Emergency triage system, Chest pain, Acute coronary syndrome, Cardiovascular risk factors

## Abstract

**Background:**

The triage of patients presenting with chest pain on admission to the emergency department uses scales based on patient clinical presentation or an electrocardiogram (ECG). These scales have different sensitivity and specificity. Although a good sensitivity allows for the prompt identification of high-risk patients, specificity prevent ED overcrowding. Moreover, ECG at triage avoids missing ST elevation myocardial infarction, which requires urgent revascularization. Our study therefore aimed to investigate whether a scale combining ECG and cardiovascular risk factors (CVRF) improves the diagnostic performance of ED chest pain triage scale.

**Methods and results:**

In this prospective single-center observational study involving 505 patients, the standard ECG-based FRENCH scale was compared to a scale combining the ECG-based FRENCH scale and the patients CVRF. The new scale was called the “modified” FRENCH. The accuracy of patient CVRF collection was evaluated by comparing the results of triage nurses and ED physicians.

Compared with the standard FRENCH scale, the modified FRENCH scale had an increased sensitivity (61% versus 75%) but a decrease in specificity (76% versus 64%) resulting in a similar diagnostic performance. Using CVRF collected by the ED physicians, the modified FRENCH scale had a sensitivity of 87% and a specificity of 56% with a significant improvement in his diagnostic performance compared with standard FRENCH scales. This improvement can be explained by an accurate collection of the CVRF by physicians compared with nurses, as suggested by the weak to moderate correlation between their respective data collection.

**Conclusion:**

In conclusion, combining ECG and accurately collected cardiovascular risks factor improves the diagnostic performance of the ECG based chest pain triage in the ED.

**Trial registration:**

Trial registration number: NCT03913767.

## Background

Chest pain may be a benign symptom or suggestive of a life-threatening condition, making it a frequent reason for emergency consultations (between 2 and 5%) [1]. However, there is a lack of consensus regarding the triage of patients with chest pain at admission in the emergency department (ED). The European and American guidelines recommend performing a 12-lead electrocardiogram (ECG) within 10 minutes after the first medical contact for patients with suspected cardiac chest pain [2, 3], which allows for the prompt identification of patients with ST-elevation myocardial infarction (STEMI) who require immediate revascularization. Patients with acute non-ST segment elevation myocardial infarction (NSTEMI) should receive continuous cardiovascular monitoring after their arrival in the ED given the risk of serious arrhythmia (ventricular fibrillation and auriculoventricular block in around 3% of patients) and undergo angioplasty within 24 hours [4]. Complications most frequently occur within the first 12 hours [5]. However, the clinical presentation of acute coronary syndrome may be atypical, as around 2 to 10% of patients have normal ECG findings [6, 7]. In the ED, it is therefore crucial to distinguish between patients requiring immediate management with intensive monitoring and those able to stay safely in the waiting room without cardiovascular monitoring, avoiding unnecessary ED crowding and ensuring a fair resources distribution among ED patients.

To help ED triage nurses in the admission triage of chest pain patients, several triage scales exist either based on clinical presentation (mainly the Manchester Triage Scale [MTS] [8], the Canadian Emergency Department Triage and Acuity Scale [CTAS] [9] and the Emergency Severity Index [ESI] [10]) or based on ECG (French Emergency Nurse Classification in Hospital scale [FRENCH]). In terms of the clinical-based scale performance for the triage of non-traumatic chest pain, a systematic review from 2017 found for the MTS a sensitivity between 70 and 80% and a specificity of 59% [11]. Concerning the ECG-based FRENCH scale, it has been validated by several studies [12, 13]. ECG-based and clinical-based scales have been compared in a prospective study from 2016 showing a similar diagnostic performance but differences in their respective sensibility and specificity [14]. Clinical-based triage was more sensitive, which reduces the waiting time for patients suffering from acute coronary syndrome (ACS). However, the lack of specificity of clinical-based triage scale may lead to an overloading of the monitoring capacity of the ED and without an ECG at triage, STEMI patients can still be allocated to a low-acuity triage score because of atypical chest pain, as it has been demonstrated in previous studies [15, 16]. Conversely, ECG-based triage was more specific and avoid missing any STEMI, although it carries the risk of overlooking patients with ACS but normal ECG.

The collection of cardiovascular risk factors (CVRF) by triage nurses could help distinguish high-risk patients from the group of patients with chest pain and a normal ECG. This study therefore investigates whether combining ECG findings and CVRF can improve the diagnostic performance of the ECG-based FRENCH triage scale in order to more accurately identify patients requiring immediate cardiovascular monitoring on admission to the ED.

## Methods

### Patient selection

The study included all consecutives patients aged 18 years and over who were admitted to the ED for non-traumatic chest pain between November 2018 and March 2020.

### Study design

This prospective single-center observational study was conducted in the ED of Saint-Luc University Hospital in Brussels, Belgium. Saint Luc University Hospital is a tertiary care hospital and has a coronary angiography center, a cardiovascular intensive care unit and a cardiac surgery department. It also provides primary care and has a dedicated ED for patients living in its area, admitting 70,000 patients per year. The study was approved by the ethics committee of the Faculty of Medicine of the Catholic University of Louvain (B403201733532, NCT03913767). The researchers adhered to research ethics and data confidentiality guidelines throughout the study.

### Determining the patient triage score and data collection

#### Triage on admission according to the standard FRENCH scale

On admission, patients with chest pain were screened according to the 2018 version of the FRENCH scale, which is routinely used in our service. The scale has five levels of severity ranging from life-threatening conditions that require the immediate management of the patient (category 1) to non-urgent situations in which the patient should be seen within 4 hours (category 5). The triage of chest pain patients according to the FRENCH scale is based on vital signs and the performance of a 12-lead ECG interpreted by a physician.

If vital signs are outside the normal range and/or if ECG is indicative of ACS, the patient will be assigned to category 1. If ECG is abnormal but not indicative of ACS and the patient experiences typical chest pain, he or she will be assigned to category 2. If ECG is normal but the patient has typical chest pain or coronary comorbidities, he or she will be assigned to category 3. If ECG is normal and the patient experiences atypical chest pain, he or she will be assigned to category 4. The respective maximum delays before medical intervention are no delay, 20, 60 and 120 minutes. For local organizational reasons, patients arriving by ambulance are moved directly to a monitoring area without undergoing triage. These patients were therefore excluded from the study.

Information about triage scores was collected from a dedicated form in the computerized medical file of patients.

#### Calculation of the modified FRENCH scale

In the modified FRENCH scales, patients with normal ECG findings were upgraded from category 3 or 4 to category 2 if they had a very high cardiovascular risk. Patients were defined as high cardiovascular risk if their Systematic Coronary Risk Estimation (SCORE) was more than 10% according to the 2016 European guidelines on cardiovascular prevention in clinical practice [17]. To estimate this risk easily and quickly, we simplified the SCORE and determined that the presence of one major or four minor risk factors was sufficient to increase the triage score. The cardiovascular risk factors are summarized in Table 1.

**Table 1:** Cardiovascular risk factors

Major	Minor
History of ischemic heart disease	Male sex
History of stroke or transient ischemic attack	Age ≥ 60 years
Diabetes	Active smoking
Lower limb arteritis	Arterial hypertension
End-stage kidney failure	Hypercholesterolemia

Triage nurses were asked to collect patients’ CVRF data on a dedicated computerized form by ticking boxes on a pre-determined list. The determination of the patients CVRF was based solely on the nurses questions about the patients medical history, without checking the patients medical record and without taking into account the severity of risk factors, the number of cigarettes smoked per day or the blood pressure measured during the triage assessment. As nurses were blinded to the calculation of the modified FRENCH score, they did not modify their ED standard of care.

#### Data collection by the physician

Physicians were asked to fill in another dedicated electronic form for the study. They collected patients’ CVRF independently from nurses. The modified FRENCH scale was also calculated based on CVRF collected by ED physicians and was called “physician” FRENCH scale.

### Final diagnosis and follow-up

The final diagnosis was determined at follow-up 30 days after the ED visit by reviewing the medical file or telephoning the patient or his or her family.

ECG interpretation and acute myocardial infarction diagnosis were based on the international definition of myocardial infarction [18]. Pulmonary embolism and aortic dissection were evidenced by computed tomography scan, pulmonary infection by biological markers and X-ray, gastroesophageal reflux by gastroscopy or symptom resolution after a therapeutic trial of gastric acid inhibiting drugs, and abdominal pathology by biological exams, imagery, or both. Musculoskeletal disorders were diagnosed if the above investigations were normal, but movement and palpation caused pain. Finally, patients with normal clinical findings, ECG, chest X-ray, and blood tests but no identifiable cause of chest pain, were categorized as “non-specific chest pain” if they did not present any event during the follow-up period.

### Statistical methods

For continuous variables, the mean and standard deviation or 95% CI were reported. The number of patients and corresponding percentages for each category were given for categorical variables. Categorical variables were compared using Pearson’s chi-square test and continuous variables using an unpaired Student’s t-test.

To evaluate the diagnostic performance of the triage system, we compared the triage score of patients with ACS (STEMI, NSTEMI, or unstable angina) with a control group of patients with chest pain of benign etiology (digestive problems, musculoskeletal pain, and non-specific chest pain). Patients with other severe symptoms or abnormal vital signs were excluded from the control group.

Receiving operating characteristic (ROC) curves were plotted to determine the precision of each triage scale in categorizing patients in the ACS or control groups. The areas under the ROC curve were compared using the method described by Hanley and McNeil [19]. Sensitivity and specificity were calculated.

The correlation between the CVRF identified by nurses and those identified by physicians was measured using Cohen’s kappa method.

All analyses were conducted using SPSS 27 software (BM SPSS Statistics for Windows, Version 27.0, Armonk, NY: IBM Corp). All *p*-values are two-sided and *p* < 0.05 was considered significant.

## Results

### Cohort characteristics

**Fig. 1:**
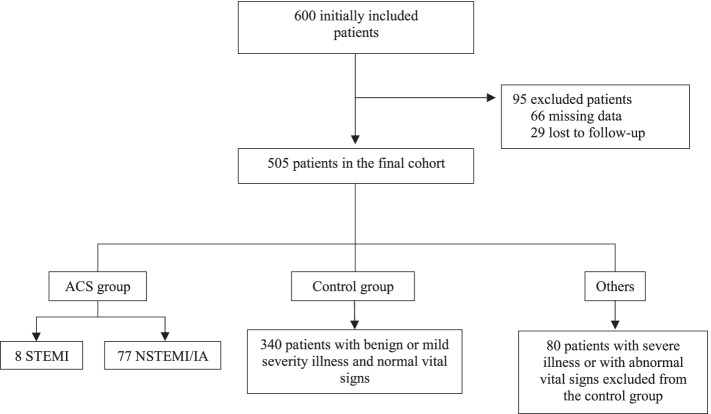
Study-selection flow diagram. Abbreviations: STEMI; ST-elevation myocardial infarction. NSTEMI; non-ST-elevation myocardial infarction. UA: unstable angina

We included 600 patients between December 2018 and March 2020. Among these 600 patients, 95 were excluded due to missing data (absence of recorded triage score) or lost to follow-up. Flow chart of patient screening and inclusion is provided in Fig. 1.

ACS was diagnosed in 85 patients, referred to as the ACS group. Among the remaining 420 patients, 340 had a benign condition or symptoms of moderate severity accompanied by normal vital signs, thus comprising the control group. Lastly, 80 patients were excluded from the control group, because their symptom severity or vital signs increased their triage category regardless of their ECG findings and CVRF.

The majority of patients were men (63%) with a mean age of 56 years. Baseline characteristics of the study cohort are summarized in Table 2.

**Table 2:** Baseline characteristics of the entire study cohort

Cohort characteristics	***N*** = 505
Mean age (years)^a^	56 ± 17
Sex ratio (M/F)	0.59
**Medical history**^b^	
Arterial hypertension	45.5% (230)
Hypercholesterolemia	40% (201)
Smoker	39.6% (200)
Ischemic heart disease	22% (110)
Diabetes	14% (70)
Lower limb arteritis	3.6% (18)
Stroke	2.8% (14)
End-stage kidney failure	1.6% (8)
**Vital signs on admission**^a^	
Heart rate (bpm)	80 ± 17
Systolic blood pressure (mmHg)	144 ± 23
Diastolic blood pressure (mmHg)	82 ± 13
Pulse oximetry (SpO_2_ %)	98 ± 4

^a^ Value expressed as mean ± standard deviation

^b^ Value expressed as percentage and number

### Final diagnosis

Most of the patients had a non-specific chest pain (38.6%). Cardiac conditions were diagnosed in 141 patients (28%). ACS was diagnosed in 85 patients (16.8%) with 8 STEMI (1.6%), 48 NSTEMI (9.5%), and 29 unstable angina (5.7%). Non cardiac diagnosis were mainly musculoskeletal pain (28.4%), gastroesophageal reflux (3.8%), pulmonary infection (2%), pulmonary embolism (1.6%) and exacerbation of chronic obstructive pulmonary disease (0.6%). The final diagnosis of patients at 30-day follow-up is summarized in Table 3.

**Table 3:** Final diagnosis at 30-day follow-up

Final diagnosis	***N*** = 505
**Non-specific chest pain**	**38.6% (195)**
**Cardiac origin**	**28% (141)**
Acute coronary syndrome	16.8% (85)
STEMI	1.6% (8)
NSTEMI	9.5% (48)
Unstable angina	5.7% (29)
Myopericarditis	3.8% (19)
Arrythmia	3% (15)
Stable angina	2.2% (11)
Cardiac insufficiency	1.2% (6)
MINOCA	0,4% (2)
Aortic stenosis	0.4% (2)
Prinzmetal angina	0.2% (1)
**Non-cardiac origin**	**33,4% (169)**
Musculoskeletal pain	24.8% (125)
Gastroesophageal reflux	3.8% (19)
Pulmonary infection	2% (10)
Pulmonary embolism	1.6% (8)
Exacerbation of chronic obstructive pulmonary disease	0.6% (3)
Pneumothorax	0.4% (2)
Aortic dissection	0.2% (1)
Pulmonary hypertension	0.2% (1)

All values are expressed as percentages and absolute numbers

*Abbreviations*: *STEMI* ST-elevation myocardial infarction, *NSTEMI* non-ST-elevation myocardial infarction, *MINOCA* Myocardial Infarction with No Obstructive Coronary Arteries

### Triage level

Using the standard FRENCH scale, 0.6% of patients were classified in category 1, 29.1% in category 2, 60.8% in category 3, and 9.4% in category 4. Using the modified FRENCH scale, 0.6% of patients were classified in category 1, 42% in category 2, 48.5% in category 3, and 8.9% in category 4. Overall, 65 patients were upgraded from category 3 or 4 to category 2 due to their high cardiovascular risk. Finally, using the physician FRENCH scale, 108 patients were upgraded from category 3 or 4 to category 2 due to their high cardiovascular risk so that 0.6% of patients were classified in category 1, 50.5% in category 2, 40.6% in category 3, and 8.3% in category 4.

### Diagnostic performance

The diagnostic performances of the standard, modified, and physician FRENCH scales are resume in Table 4. They were compared using the area under the ROC curve (AUC). Significant differences in AUC were found between the standard and modified FRENCH scale compared to the physician FRENCH scales (0.689 [0.624–0.754] and 0.697 [0.638–0.757] versus 0.717 [0.663–0.771] respectively, *p* < 0.001).

**Table 4:** Diagnostic performances of the standard, modified, and physician FRENCH scales

	Standard FRENCH scale	Modified FRENCH scale	Physician FRENCH scale
Sensitivity %	61	75	87
Specificity %	76	64	56
Positive predictive value %	34	29	28
Negative predictive value %	91	93	95
AUC*	0.689 (AUC 1)	0.697 (AUC 2)	0.717 (AUC 3)
Lower limit 95% CI	0.624	0.638	0.663
Upper limit 95% CI	0.754	0.757	0.771
	AUC 1 vs AUC 2	AUC 2 vs AUC 3	AUC 1 vs AUC 3
*p*-value	*p* = 0.86	*p* = 0,02	*p* < 0.001

*Area under the ROC curve

### Correlation between the CVRF collection by triage nurses or physicians

The correlation between the CVRF collected by triage nurses on the one hand and by physicians on the other is presented in Table 5. Correlation was weak to moderate at best, with a Cohen’s kappa coefficient between 0.31 and 0.56.

**Table 5:** Correlation between the cardiovascular risk factors identified by nurses and physicians

Variable	Phycisians*	Nurses*	Cohen’s kappa	95% CI
Arterial hypertension	275	110	0.38	(0.31–0.45)
Hypercholesterolemia	201	78	0.31	(0.23–0.39)
Smoking	124	64	0.49	(0.39–0.58)
Cardiac history	110	66	0.55	(0.46–0.64)
Diabetes	70	34	0.55	(0.43–0.67)
Arteritis	18	8	0.37	(0.13–0.61)
Stroke/transient ischemic attack	14	7	0.56	(0.3–0.81)
End-stage kidney failure/dialysis	8	4	0.32	(0–0.67)

*Value expressed as the number of patients

## Discussion

This prospective study of 505 patients admitted to the ED compared an ECG-based chest pain triage scale (standard FRENCH scale) to a triage scale combining an ECG with CVRF (“modified” or “physician” FRENCH scale) and support the use of CVRF to improve chest pain patient triage. The standard FRENCH scale had a diagnostic performance of 0.689 (0.624–0.754), with a sensitivity of 61% and a specificity of 76%, corroborating a previous study [14]. Both the modified and the physician FRENCH scales had an increased sensitivity and a reduced specificity, but only the physician FRENCH scale finally improved his diagnostic performance.

The characteristics of a triage system should be chosen according to the capacity of the emergency department, balancing the objective of not missing an ACS with the objective of not saturating monitored beds, to ensure an equitable distribution of resources. Increased sensitivity prevents missing ACS patients, but is only affordable for EDs with sufficient resources to admit more chest pain patients quickly.

The difference between the modified and the physician FRENCH scales can be explained by the inappropriate collection of data on CVRF by the triage nurses compared to physicians, since the correlation between their respective collection of CVRF was poor and since medical record can in this situation certainly be used as the reference. Actually, this weak correlation was due to numerous risk factors not recorded by nurses at triage. There are several possible reasons for this, the triage nurses could have been less rigorous but so could lack of time, overcrowding, asking basic questions of patients instead of further anamnestic investigations and the fact that triage is done without opening patients medical records. An appropriate nurse education and training in the collection of CVRF and the use of electronic checklists could help to accurately collect patients CVRF which is of paramount importance, as shown by the performance of the physician FRENCH scale. Moreover, the effective use of the modified scale would probably better encourage triage nurses to collect all CVRF than his use in the context of a study.

A recent study on the evaluation of chest pain triage showed that the use of the Emergency Department Assessment of Chest Pain Score (EDACS) has a better diagnostic performance than the Manchester Triage System [20]. The EDACS score is a simple tool combining cardiovascular risk factors and clinical features of chest pain. This study therefore supports our findings on the benefit of using CVRF collection at triage. However, as our study points out, the routine use of a more sophisticated score by triage nurses would require significant additional resources and further investigations should be made to find the most effective scoring system.

This study has several limitations. First, the exclusion of patients arriving by ambulance lead to a low rate of STEMI patients in the study (9,4% of the ACS group), considerably lower than what is observed in other studies (27–30% of the ACS group) [21, 22]. A higher rate of STEMI patients would have led *de facto* to a higher specificity of an ECG-based triage system. Second, we had a large number of patients excluded due to missing data, mainly the absence of recorded triage score or the impossibility to contact the patients for the 30-day follow-up.

## Conclusion

In conclusion, the sensitivity of ECG-based chest pain triage system in emergency departments can be increased by using cardiovascular risk factors to triage patients with normal ECG findings. More accurate collection of cardiovascular risk factors could increase the diagnostic performance of the triage system. This study highlights the challenges of implementing a more sophisticated triage system, which requires sufficient resources and appropriate education and training.

## Data Availability

All the data of the study are available on request at the following address: melanie.dechamps@saintluc.uclouvain.be
